# A New Approach to Integrate Internet-of-Things and Software-as-a-Service Model for Logistic Systems: A Case Study

**DOI:** 10.3390/s140106144

**Published:** 2014-03-28

**Authors:** Shang-Liang Chen, Yun-Yao Chen, Chiang Hsu

**Affiliations:** 1 Institute of Manufacturing Information and Systems, National Cheng Kung University, Tainan City 70101, Taiwan; E-Mail: slchen@mail.cjcu.edu.tw; 2 Department of Business Administration, Chang Jung Christian University, Tainan City 71101, Taiwan; E-Mail: chiangh@mail.cjcu.edu.tw

**Keywords:** Software-as-a-Service, Internet of things, Logistic Cloud, cloud manufacturing

## Abstract

Cloud computing is changing the ways software is developed and managed in enterprises, which is changing the way of doing business in that dynamically scalable and virtualized resources are regarded as services over the Internet. Traditional manufacturing systems such as supply chain management (SCM), customer relationship management (CRM), and enterprise resource planning (ERP) are often developed case by case. However, effective collaboration between different systems, platforms, programming languages, and interfaces has been suggested by researchers. In cloud-computing-based systems, distributed resources are encapsulated into cloud services and centrally managed, which allows high automation, flexibility, fast provision, and ease of integration at low cost. The integration between physical resources and cloud services can be improved by combining Internet of things (IoT) technology and Software-as-a-Service (SaaS) technology. This study proposes a new approach for developing cloud-based manufacturing systems based on a four-layer SaaS model. There are three main contributions of this paper: (1) enterprises can develop their own cloud-based logistic management information systems based on the approach proposed in this paper; (2) a case study based on literature reviews with experimental results is proposed to verify that the system performance is remarkable; (3) challenges encountered and feedback collected from T Company in the case study are discussed in this paper for the purpose of enterprise deployment.

## 1. Introduction

For companies that have their own information technology (IT) departments, a common issue is that different production information can have different usages. For example, new products may need to be qualified before export due to unstable production recipes. Therefore, the IT department needs to develop software for on-line production use. Companies that do not have their own IT departments must pay large maintenance fees for their production applications. Traditionally, it has been very difficult, if not impossible, to create a connection between the business layer and the production layer at the bottom (shop floor) because they are mainly based on different interfaces and technologies [1]. By taking advantage of service orientation software architecture, automation software can be composed and orchestrated in a service cloud [2]. Moreover, information access, retrieval, and processing may be carried out in different organizations, allowing information support to be heterogeneous. Based on web service technology and service-oriented architecture (SOA), cloud systems can be designed and implemented in real process flow. Software-as-a-Service (SaaS) inherits the advantages of SOA, and is categorized by the National Institute of Standards and Technology (NIST) into mature SaaS architectures. Mature SaaS applications have the following properties:

- Scalability: capable of handling growing amounts of work in a graceful manner.
- Multi-tenancy architecture: one application instance may serve hundreds of companies, in contrast to host applications where customers are each provisioned their own server running one instance of the software.
- Metadata-driven configurability: instead of customizing the application for a customer (requiring code changes), the user can configure the application using metadata.

The present study provides a design for a logistic cloud framework maturity level 3. SaaS maturity level 3 adds scalability through a multi-tier architecture that supports a load-balanced farm of identical application instances that run on a variable number of servers. The provider can adjust system capacity to match demand by adding or removing servers without further altering the software architecture [3,4]. The level 3 maturity model can serve a huge number of tenants with one scalable software infrastructure. Salesforce.com entered the market with this maturity model based on the concept of multi-tenants model.

The Internet of things (IoT) is a paradigm that takes advantage of sensor networks. It is rapidly gaining ground in modern wireless communications, with its position and status known, where services and intelligence are added to this expanded Internet, fusing the digital and physical worlds. The basic concept is the pervasive presence of objects, such as radio-frequency identification (RFID) tags, actuators, mobile phones, and sensors [5]. The benefits of IoT to developing and emerging economies are significant, and strategies to realize these need to be found. As a result, applying IoT for modeling logistic system is a promising solution for researchers [6]. For the purpose of developing an IoT environment, sensor networks are often considered as the major technology [7]. In real logistic environments in enterprises, sensors are used for both logistic inventory and pallet inventory in many business processes. Commonly adopted sensors include global positioning system (GPS) sensors, barcode sensors, RFID sensors, and mobile devices. IoT will fundamentally change the supply chain process and management means for new development opportunities in the areas of enterprise manufacturing and supply chain management [8]. In this paper, a logistic cloud framework is proposed based on SaaS cloud computing and IoT. The framework is a cloud computing paradigm for system developers and decision makers for manufacturing industries. We discovered some weaknesses of the current logistic systems:

- Current systems are usually built case by case to fit certain logistic environments.
- Traditional logistic systems lack reusability and flexibility.

There are three main contributions of this research:

- A Logistic Cloud based on SaaS and IoT is proposed based on our previous published work [9].
- A logistic System for Logistic Management based on Logistic Cloud is deployed and applied to a case study scenario in T Company with performance experiment.
- A design with its own design patterns and a scenario case study are described in a normal logistic environment to prove that the SaaS Logistic Cloud is workable in the actual logistic environments of enterprises.

After implementing the proposed framework with a prototype system developed in previous research by these authors, T Company provided feedback that indicated that the architecture has numerous advantages:

- Ease of installation and configuration.
- Combination of ICT technologies for manufacturers.
- Low cost and pay-per-use.
- Ease of connection and integration.
- Customizable access to other applications.
- Fits various logistic scenarios.
- Centralized and easy to maintain.

## 2. Related Works

### 2.1. Cloud Manufacturing

According to the Market Intelligence & Consulting Institute, the global market for SaaS cloud computing has reached about 1.47 billion US dollars. According to Dreamsimplicity.com, the fastest growing SaaS firms are SuccessFactors, Zuora, Marketo, Boomi, SpringCM, AdaptivePlanning, NETSUITE, PLABMiLL, FreshBooks, and Coupa. Most of them are providers of management systems for companies. The services provided by SaaS logistic vendors are listed in Table 1 (from Gartner Inc., Stamford, CT, USA). The table implies that investments in SaaS software have increased tremendously. The authorities for Chinese ICT research institutions (CCW Research) recently stated that SaaS software has reached 160 billion NT dollars in 2011. Thus, it is obvious that SaaS cloud computing business information systems help enterprises develop. Cloud manufacturing is a computing and service-oriented manufacturing concept developed from existing advanced manufacturing models, architectures, and enterprise information technologies under the support of IoT, service computing, virtualization, and advanced computing technologies. It aims to virtualize different manufacturing resources for on-demand use and provide service-oriented on-demand services on the cloud. Xu [10] defined cloud manufacturing as “a model for enabling ubiquitous, convenient, on-demand network access to a shared pool of configurable manufacturing resources that can be rapidly provisioned and released with minimal management effort or service provider interaction”. Many researchers have proposed the idea of applying cloud computing to transform manufacturing [11–15]. Some others have proposed that service-oriented architectures in cloud computing can coordinate different platforms more flexibly. Researchers have shown that manufacturing systems combined with cloud computing is workable and remarkable in the supply chain process. The results are summarized in Table 2.

### 2.2. IoT Technologies Used for Logistic Systems

IoT uses RFID, infrared sensors, GPS, laser scanners, and other information sensing devices, according to the agreed protocol, to any article connected to the Internet to provide information exchange and communication in order to intelligently identify, locate, track, monitor, and manage a network [26]. RFID tags can be used for coordinating data with their ability to attach on different surfaces of objects. They can be sensed in real-time using active or passive RFID readers. RFID technologies are widely used in many industries, such as construction, education, manufacturing, healthcare, and in airline industries [27]. In addition to active/passive RFID, modern technologies such as information pushing, Tablet PCs, Wi-Fi, 3G networks, mobile devices, smart phones, Google maps and service oriented architecture have become a new area of research for constructing applications and applying solutions to traditional manufacturing. However, such technologies have seldom been applied to logistic industries since the infrastructure is usually lacking in traditional manufacturing IT systems. Some industries even lag behind other industries in regard to communication and information technologies [28]. Currently, the development of information systems in industries such as manufacturing and logistics is usually conducted case by case, preventing reuse and rebuilding. With the development of web services and the implementation of SOA, departments in companies can componentized their information and services through software componentization in an unprecedented way [29]. According to Forrester Research Institution RFID middleware has the following four functions [30]:

- Reader coordination: end users can use RFID middleware for loading, monitoring, deploying, and sending commands to the reader. For example, some middleware manufacturers provide hot-plugging functions, allowing users to operate dynamically.
- Data filtering and aggregation: when tag reading errors occur, the responsibility of RFID middleware is to fix the errors by implementing correction algorithms. When dealing with large amounts of data, RFID middleware must provide buffering to filter and aggregate data.
- Data routing and integration: some companies have their own SCM, ERP, and CRM systems. Such companies hope that RFID middleware can be equipped with data routing and integration functions, which can enhance operations utilizing RFID.
- Process management: RFID middleware must be capable of data monitoring and data aggregation. Management should be notified when inventorying is not adequate.

Researchers have shown that systems combined with IoT technology, such as RFID, Zigbee, and other sensors, can help improve the collection, sharing, and exchange of information in the supply chain process. The results are summarized in Table 3.

## 3. IoT-Based Framework for Logistic Operation Process

Scenario-supported approaches enable innovation managers to align the development process of new products or services according to new criteria and to predict and test market capability with regard to implementation opportunities. Moreover, this step can reduce the risk of failure due to inappropriateness for the market. The present study cooperated with a manufacturing company (T Company) to survey the business processes of logistics in enterprises. To study the business process of logistics in traditional manufacture enterprises, some sample diagrams were used for analyzing the processes in real logistic environments. The business process was studied to determine the real work flow in a logistic company, which is important for cloud services that need to be developed in the logistic cloud. In this section, some logistic processes (pallet inventory, pallet monitoring, and global logistic tracking) are studied for the purpose of deploying IoT technologies. These technologies are used for developing cloud-based services in the proposed logistic cloud discussed in Section 4.

### 3.1. RFID/Zigbee-Based Pallet Inventory

**Figure 1.**
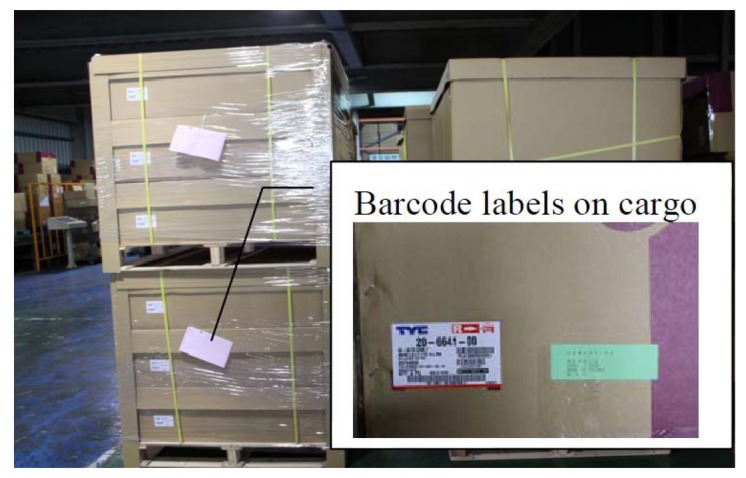
Traditional cargo tagged with dispatch list. Some of them are barcode-enabled, but others are not (as-is).

The pallets in T Company carry cargo that is ready to ship to third parties. Barcode tags are attached to the cargo, which enables staff to inventory all the goods that are ready for shipping, as shown in Figure 1. Sometimes cargo goes missing due to unpredictable issues, such as incorrectly or incompletely filled out worksheets. Cloud services with long-distance RFID for pallet inventory are proposed to solve these problems. Some functions were suggested by researchers based on Table 2 for us to develop with cloud services:

- Functions for reading barcode IDs.
- Functions for storing barcode IDs.
- Functions for connecting barcode IDs.

### 3.2. RFID/Zigbee-Based Pallet Monitoring

In T Company, pallets are moved using a conveyer inside the factory. The current position of the pallets and what goods are on the pallets is important information. Traditional systems for monitoring pallets rely on barcode tags on the goods. This study adopted RFID technology for monitoring the goods and pallets more precisely and quickly. Monitoring services for monitoring pallet position is another key cloud service that needs to be considered for the logistic cloud. They include:

- Functions for using RFID to monitor pallets and goods on moving conveyers.
- Functions for using IP cameras to monitor pallets and goods inside factories.
- Functions for notifying staff about the condition and information related to pallets and cargo.

### 3.3. Location-Based Logistic Transportation

**Figure 2.**
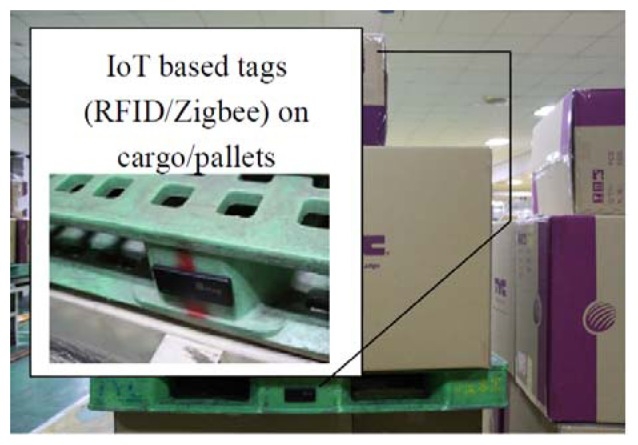
IoT-technology-enabled logistic scenario. RFID and barcode tags on cargo at the same time (to-be).

**Figure 6.**
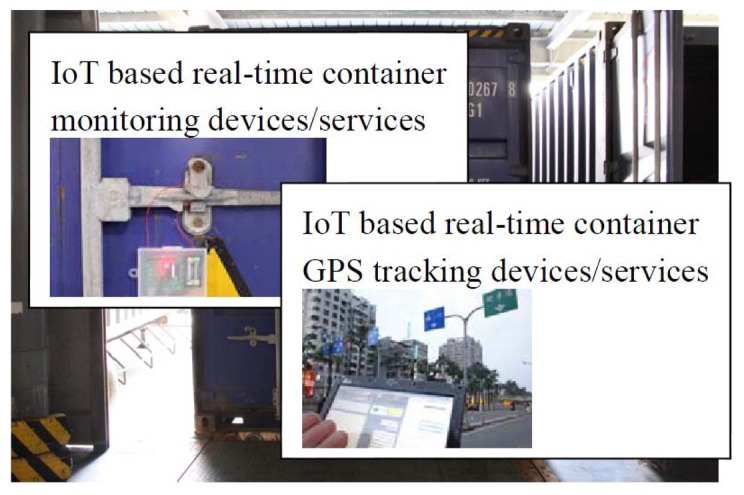
IoT-based container seal development and deployment in real environment.

Pallet inventory and pallet monitoring are regarded as indoor shipping. Outdoor shipping by T Company is also considered here. Pallets and goods in T Company are inventoried by staff, and the data is stored in the database of T Company. All the goods are sealed and put into containers, as shown in Figures 2^3^, 4 and5 and 6. All containers are shipped to a third party at a certain time. Therefore, the tracking of goods is important for both T Company and its third parties. Traditionally, containers are sealed using disposable container locks. Once the container is sealed, all the information is written on a shipping list. The list contains a series of numbers, which mapped to the disposable container seal shown in Figure 6. When the container arrives at the destination port, the staff unseals the lock by breaking the lock. Some issues related to this process are:

- Broken seals are not reusable. This may cause environmental and recycling issues.
- Disposable seals are designed only for sealing and can't be located during transportation.
- Container security is an important issue.
- Container shipping enterprises waste human resources on container inventory.

From a study of the outdoor shipping operation process of T Company, the following functions are needed in the logistic cloud for outdoor shipping:

- Functions for GPS tracking of containers.
- Functions for coordinating electronic maps.
- Functions for accessing information with cloud databases.

## 4. Case Study: Design of Logistic Cloud Based on IoT and SaaS Cloud Computing

### 4.1. Design of Logistic Cloud Framework

**Figure 7.**
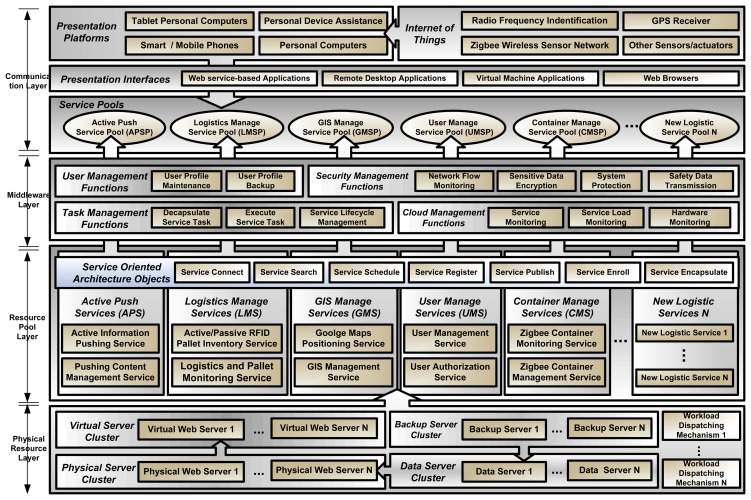
Framework of Logistic Cloud.

In order to explore and implement the concept of SaaS and IoT integration, a Logistic Cloud Framework is proposed in this research by referencing our previous studies [41,42]. The Logistic Cloud proposed is used to explain the framework, model and concept in the case study conducted in this research. The framework of the logistic cloud (see Figure 7) is divided into four layers, namely a Communication Layer (CL), a Middleware Layer (ML), a Resource Pool Layer (RPL), and a Physical Resource Layer (PRL).

- Communication Layer (CL): The CL allows the system to encapsulate cloud computing into standard functions, such as service connection, service enrollment, service searches, service visits, and service scheduling. In common developmental environments, the key technologies are the ML and RPL.
- Resource Pool Layer: The RPL integrates existing types of sources and web services into service pools, such as a Pallet Inventory Service Pool, a Pallet Monitoring Service Pool, and a Logistic Transportation Service Pool, for enterprises and clients to access through CL. Services can be abstracted by the system developers with different platforms when applications are designed. A resource represents all available services that encapsulate all operation flows involved or those assisted by technology components. For example, barcode readers are often used for assisting the operation flow of cargo inventory. Data collection is triggered by software. A resource pool encapsulates the software as a service, which is possible for all new designed systems to rebuild as a new function.
- Middleware Layer: The ML is responsible for handling tasks such as User Management (UM), Task Management (TM), Resource Management (RM), and Security Management (SM). The ML can be regarded as a direct interaction interface between the end user (service requester) and service provider (resource pool). The commands of the service requester are directly transmitted through the intranet. UM is used to implement the business model in cloud computing, including user management, user environment deployment, user data exchange and management, and user logging. TM is responsible for executing and managing applications requested by users, including call service tasks, execution of service tasks, service lifecycle management, and service deployment and management.
- Physical Resource Layer: The PRL is responsible for data storage and the software/hardware infrastructure, including computers, databases, network equipment, storage, and software. The physical resource environment in this study had five individual servers, or five service pools.

**Figure 8.**
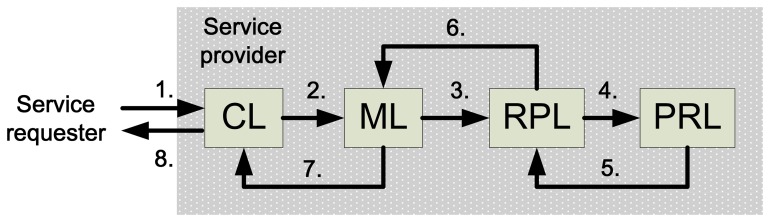
The interaction process flow for accessing the logistic cloud.

The interaction process flow for accessing the logistic cloud is shown in Figure 8. The interaction process is as follows:

- Service requester requests services in the logistic cloud. A service requester is users in real business process models, which can be a member of the staff, a manager or a third party vendor in a logistic company.
- The CL receives the request and passes it through XML in a standard SOA protocol. Request packets are encapsulated and transformed into XML data type. Users can access the services on the Logistic Cloud with different presentation interfaces, which shows that the Logistic Cloud is capable of dealing with cross platform requests.
- The ML is responsible for checking the authorization of the service requester and determines whether the request is legal. If the request is an attack request, it blocks the access rights. For example, DDoS and SQL injection requests are illegal requests. If the requests are legal, they are passed to RPL. Some other new internet attacks will be defended with the updating of the software and infrastructure constructed in the proposed Logistic Cloud.
- The RPL is encapsulated with service pools. It provides the service that the requester needs and acquires the data from the PRL. Service-oriented architecture is implemented in this layer. Logistic business processes are merged in the services provided in the Logistic Cloud within this layer. New services and functions are able can be expended and ported into this phase.
- The PRL will do the second stage data filtering. All the data requests from the RPL are buffered and queued in this layer. This layer is responsible for handling the data in a first-in-first-out phase. When the PRL respond s to the right source, the source is returned to the RPL.
- The RPL transforms the service and the source from the PRL to the ML. The ML logs the entire process and passes the final result to the service requester through the presentation platforms in the CL. Therefore, IoT devices must be compatible with presentation platforms adopted by service requesters.

### 4.2. Service-Accessing Platforms of Logistic Applications

Logistic cloud services can be implemented with either direct or indirect access. Direct access means that all the services are abstracted through the logistic cloud (no third-party service providers). In other words, direct access implements all services from the logistic cloud. However, some companies have many sub-enterprises, so they may not want to redevelop their services. Therefore, services inheriting different software platforms have become another way to access services in the Logistic Cloud, and are regarded as indirect access through the cloud. Platforms supported by proposed logistic cloud is listed in Table 4.

### 4.3. Service Access Model of Logistic Applications

**Figure 9.**
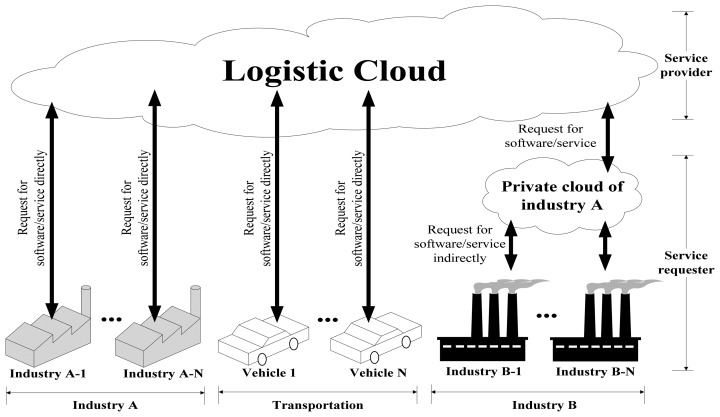
Service access model.

**Figure 10.**
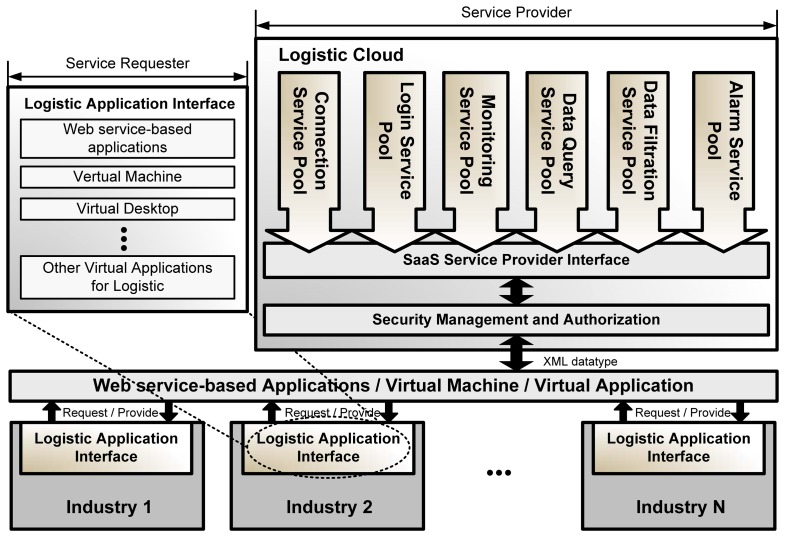
Service access model.

An Internet connection is a fundamental requirement. All services in the logistic cloud are modular, which may lead to platform compatibility issues. Coordinating agents in different enterprises are thus needed. Coordinating agents are capable of communicating and interpreting various devices used in companies. For example, some companies use RFID for inventory, whereas others use barcodes. The coordinating agents are responsible for collecting and re-decoding the messages from equipment. Detailed service access model applications and a description of Figure 9 are provided in Figure 10.

Different industries are able to access the service provider (Cloud Supplier) by implementing their logistic application interfaces in a common SaaS Service Provider Interface (SPI). An SPI is usually regarded as an API or as another common standard interface. After the security management and authorization, users can access the Logistic Cloud with Web service-based systems, virtual machines, or virtual applications, which are described as below:

- Web service-based systems: Systems developed by abstracting Web services on the Logistic Cloud with comment SPIs. Industries that have their own IT departments have the ability to develop their own industrial systems without outsourcing. Graphical user interfaces can be designed on a case by case basis to meet industry demand.
- Virtual machines: Virtual machines have the ability to be controlled and deployed on demand by users by implementing the systems on the virtual machines in the logistic cloud. Software can be used and accessed through remote applications such as VMWare vSphere or Microsoft SCVMM.
- Virtual applications: Cloud systems or services can be accessed through virtual applications on the Internet without the limitation of operating systems or computers. For example, products such as Thin Client are commonly used by enterprises for remote control and work. This enables another work process for current cloud applications. Therefore, different system interfaces have been developed with different platforms. Services that are the same are abstracted by developing with different platforms.

## 5. Performance Evaluation of Case Study

### 5.1. Equipment Performance Evaluation

In regard to RFID equipment tests, we stick an RFID label to the simulated pallet and deploy the reading devices at steps in each area, push the goods into the gate, and set up the stock check interval. We construct an RFID long-distance stock-checking system to test the RFID's repeated stock checking at the pallet. Since we hope the pallet can be precisely sensed after passing the gate, we set 20 reading times as the testing unit, and 100, 200, 500, 1000 ms as the repeated reading unit. The testing results are listed in Table 5:

From the experimental data, we can generalize that the larger the reading interval, the less accuracy for the repeated reading. Therefore, when deploying in reality, the reading interval should be reduced to less than 200 ms to count the pallets passing the gate for ensure the precision of pallet data acquisition.

In this research, Zigbee was adopted because of its limitless transmission and I/O integration functions, and a wireless container monitoring system was developed. The device automatically sends back monitoring messages periodically. When the locking point of the device is destroyed or intruded upon, it will send back the message to the administrator the container's no. When it comes to the equipment test of the container's electronic lock, in the experimental stage, we installed a Zigbee electronic lock on the general container with 20 times as the testing unit, and 100, 200, 500 and 1000 ms as the unit to proceed with repeated reading. The testing results are listed in Table 6.

From the experimental data, we can generalize that the larger the reading interval, the more accurate is the repeated reading. Therefore, in real deployment, if we want to count the pallets passing the gate using Zigbee, the reading interval should be increased to more than 500 ms.

### 5.2. Web Server Performance Evaluation

According to the testing results based on the experimental environment depicted in Table 7, regardless of whether the web servers are physical or virtual web, the average response time rises along with the log-in user number. As for comparison of efficacy, Physical web server = Virtual web server (1 G) > Virtual web server (512 MB), with the time difference controlled within 0.002 s. From Table 8, we can infer that the more connection users, the bigger difference there will be in regard to operation between a virtual web server (RAM: 512 MB) and a physical web server. As a result, we set the testing result and the upper user limit of 4,000 as a basis by which to calculate the efficacy. Under the same conditions, we can further generalize the information listed in Table 8 and determine that if this research deploys using a physical web server, within 7.381 ± 1.000 ms, there are 4,000 users connecting to the Web for identification; if this research deploys using a virtual web server, within 8.648 ± 1.000 ms, there are 8,000 users connecting to the Web for identification. We can therefore compute that with the same software/hardware equipment, the user number that the service can provide per ms increases at least 1.7 times. This proves that for a cloud service provider, offering a virtual web server for an enterprise to rent can raise the server's efficacy.

## 6. Challenges of Applying Logistic Cloud and IoT to Logistic Enterprises

### 6.1. Deployment Challenges

There are four types of cloud deployment models, namely public, private, community, and hybrid clouds. Each cloud model is suitable for certain logistic enterprises. The public cloud is based on the concept of sharing services and infrastructure provided by a third-party service provider. The private cloud is based on the concept of sharing limited data within a certain organization. The hybrid cloud consists of both private and public clouds. Therefore, selecting the most appropriate infrastructure is a key issue. For example, business production information is often confidential. Thus, the private cloud is best for such enterprises. However, maintenance problems might arise since a team is required to maintain a private cloud.

### 6.2. Security Challenges

Enterprise information often contains information about customers and employees. Some of the information contains private data that must be protected. Therefore, data and information security issues are fundamental concerns for enterprises when building their own clouds. Some research has shown that information security (IS) issues are a novel problem that is common and must be urgently solved. In companies, the deployment is often based on the supervisors' awareness of threats. According to Kankanhalli *et al.*, preventive efforts include deploying advanced security software or controls to protect IS assets, such as advanced access control, intrusion detection, firewalls, surveillance mechanisms, and the generation of exception reports [43].

### 6.3. Staff Training Challenges

In some traditional manufacturing companies, staffs lack the ability to adopt new technologies such as RFID and GPS. Training staff to use new technologies is thus an important issue. A comprehensive training course is a basic solution for enterprises that want to deploy cloud-based systems.

### 6.4. Business Process Challenges

Traditionally, business process management systems provide functions that interact with business processes. For T Company, flexibility, deplorability, and affordability are urgent concerns. For example, RFID tag cost may not be an issue for high-profit products, but can be an issue for low-profit products. Moreover, other issues such as server farm building and the fundamental infrastructure of cloud computing should be considered.

### 6.5. Organizational Structure of Enterprises

In manufacturing companies, systems are separated into sub-systems. They are not normally connected to each other. Some systems store data in separate databases. This may cause maintenance problems. For example, different machine tools may have different types of commands. System developers may spend a lot of time and money merging all these commands by implementing command middleware or agents. Therefore, determining methods by which to merge all the functions and to increase the flexibility of systems are highly important goals that decision-makers and computer programmers must solve. The manufacturing cloud is separated into many aspects based on enterprise organizational structure. Different services must fit different departments. For example, services for human resources are different from those for accounting. The fundamental structure of a manufacturing cloud should be based on this concept.

## 7. Conclusions

This study proposed a framework with a four-layer model for enterprise IT departments to design their own logistic systems based on SaaS cloud computing and IoT technology. From a study of business processes and current systems in T Company, some challenges that developers and enterprise decision-makers need to consider before developing a cloud-based system based on the proposed logistic cloud were identified. The discovered challenges are a reference for developers and researchers. Feedback collected from T Company is listed in Table 9. There are three main contributions in this paper: (1) enterprises can develop their own cloud-based logistic management information systems based on the approach proposed in this paper; (2) a case study based on a literature reviews with experimental results is proposed to verify that the system performance is remarkable; (3) challenges encountered and feedback collected from T Company in this case study are discussed in this paper for enterprise deployment.

## Figures and Tables

**Figure 3.**
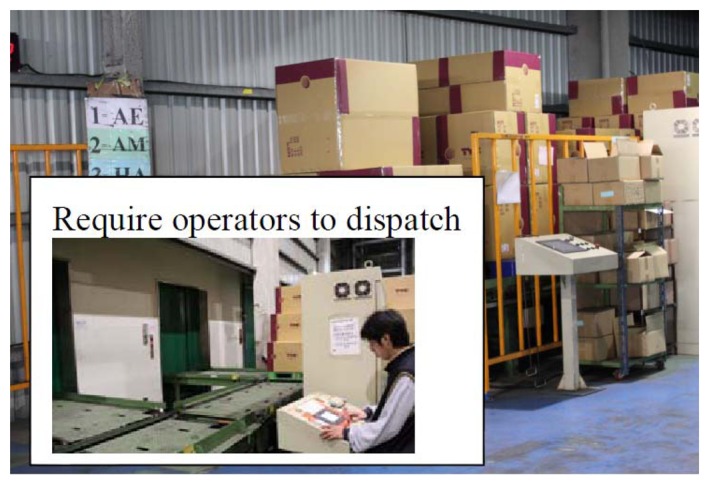
Pallets and goods on the conveyer. Operators are required to dispatch cargo into categories (as-is).

**Figure 4.**
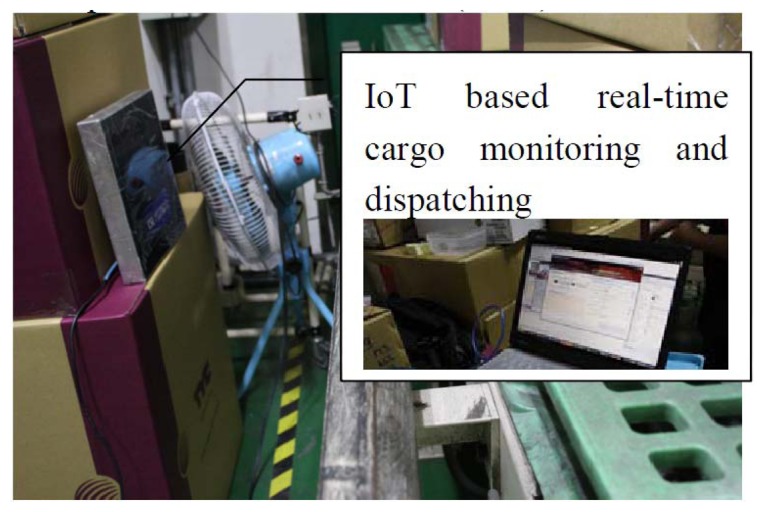
IoT-technology-enabled real-time cargo monitoring and dispatching through computers and work stations (to-be).

**Figure 5.**
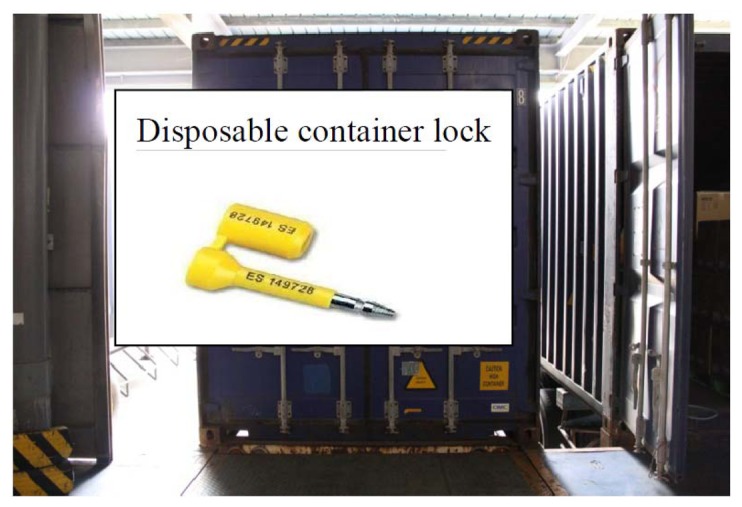
Photograph of disposable container lock.

**Table 1.** Types and output value of SaaS logistic services on the global market. (Billion USD).


**No**	**Types of SaaS Logistic Services on the Market**	**2009**	**2008**

1	Content, Communications, Collaboration (CCC)	1664	1385
2	Office Suites	44	36
3	Digital Content Creation (DCC)	40	28
4	Customer Relationship Management (CRM)	1,475	1,211
5	Enterprise Resource Planning (ERP)	802	761
6	Supply Chain Management (SCM)	534	459
7	Other Application Software	305	250
8	Total Enterprise Software	4,865	4,133

Source: Gartner (2010)			

**Table 2.** Summary of cloud manufacturing previous studies.

**Study**	**Notes**
Laili *et al.* [16]	Proposed a computing resource allocation cloud manufacturing framework (CMfg) and designed a highly intelligent algorithm for optimal allocation of computing resources in CMfg. The research provides a new model which can enhance the inefficiencies in service-oriented manufacturing.
Tao *et al.* [17]	Described the relationship between cloud computing and CMfg. A computing and service-oriented model with a detailed description and model is proposed in this research using the support of IoT, and advanced computing virtualization and service-oriented technologies are proposed.
Tao *et al.* [18]	A parallel algorithm for solving large-scale software and hardware cloud services is proposed. Compared with traditional serial intelligent algorithms and classical parallel intelligent algorithms, the results are remarkable and can be applied to other large-scale composition service networks.

Xu [19]	This research discusses some of the essential features of cloud computing and two types of cloud computing adoptions in manufacturing and cloud manufacturing. An interoperable and flexible cloud manufacturing system (ICMS) is proposed to provide users with a big range of flexible manufacturing capabilities.
Zhang *et al.* [20]	A CMfg prototype and the existing related works conducted by the authors' group on CMfg are briefly presented. Through taking virtual machine mappings as the accessing carrier, distributed resources are mapped into virtual resources (virtual machine). Several function modules are mainly achieved through related technologies.
Wu *et al.* [21]	A unique strategic vision for cloud manufacturing is documented. Comparison of the strategy vision and current state leads to suggestions for future work. Some potential impacts and future concepts for research are also discussed in this review.
Putnik [22]	An introduction to the development concept of ubiquitous and cloud manufacturing is presented. Architecture through an informal and conceptual presentation of cloud manufacturing is also discussed, which enables development of an advanced manufacturing system or enterprise on different complexity levels.
Chen *et al.* [23]	An innovative technology of virtual COM port technology is proposed in this research. A prototype system is addressed in this paper to implement the concept of service-as-a-software cloud computing concept.
Giriraj *et al.* [24]	This paper establishes the value of realizing cloud connects and usage state of affairs in the cloud manufacturing environment. It offers monitoring vision and control and a case study with the help of a manufacturing execution assembly system. The purpose of the theory part of the study is to first introduce the concept of cloud connect in the respective field of a manufacturing execution assembly system.
Hung *et al.* [25]	A cloud computing-based equipment monitoring system (EMS) for the CNC machine tool industry to illustrate the paradigm shift of EMSs from basing on the Internet to basing on the cloud. Intended to overcome the shortcomings of traditional web GUIs. This paper proposes a novel web application implementation framework with cloud computing architecture and completes the testing operations.

**Table 3.** Summary of IoT technology studies used in industries.

**Study**	**IoT Sensor**	**Notes**
Jaselskis *et al.* [31]	RFID	RFID technology that enhances the operation of industries was proposed.
Yagi *et al.* [32]	RFID	Product information can be handled through its own RFID tags.
Ergen *et al.* [33]	RFID	RFID technology can be designed as a mobile infrastructure for locating components.
Wang *et al.* [34]	RFID	RFID scanning and data entry mechanisms and personal digital assistants were integrated, and a web-based system called RFID-QIM was developed to improve the acquisition of quality inspection data in material test labs.
Chow *et al.* [35]	RFID	A system for a warehouse operation environment that enhances the effectiveness in formulating resource usage package and managing resource operation was proposed.
Zhao *et al.* [36]	RFID	An architecture that supports different readers was proposed. An SOA-based architecture for large-scale deployment and integration of RFID devices at the edge of a network was proposed.
Kwok *et al.* [37]	RFID	The feasibility and practicality of shifting the focus of product identification from traditional human readable or kiosk-based solutions was explored.
K. Y. Lu [38]	Zigbee	Zigbee technology was used to develop a system that uses a wireless approach to improve conventional work orders for high information transparency, automatic data capture, and instant data transmission.
Garcia *et al.* [39]	Zigbee	Zigbee technology was used to monitor humidity and various cargo densities.
Yu *et al.* [40]	Zigbee	A ZigBee-wireless-network-oriented shop floor capable of effective remote monitoring was constructed.

**Table 4.** Platforms supported by proposed logistic cloud.

**Supplier**	**Product**	**Platform**	**License Type**	**Logistic Cloud Access**
Microsoft	Hyper-V	Windows NT	Proprietary	Yes
VMware	VMware Infrastructure	Windows NT/Linux	Proprietary	Yes
Citrix	XenServer	Linux	Proprietary	No
Oracle	Virtual Box	Linux	Open Source	Yes
SWsoft	Open VZ	Linux	Open Source	Yes
Virtual Iron	Virtual Iron XEE	Linux	Proprietary	Yes
N/A	QEMU	Linux	Open Source	No

**Table 5.** RFID reading experiment results.

**No.**	**Reading Interval (ms)**	**Reading Times**	**Success Times**	**Failure Times**
1	1,000	20	6	14
2	500	20	13	7
3	200	20	19	1
4	100	20	20	0

**Table 6.** Zigbee reading experiment results.

**No.**	**Reading Interval (ms)**	**Reading Times**	**Success Times**	**Failure Times**
1	1000	20	20	0
2	500	20	20	0
3	200	20	15	5
4	100	20	9	11

**Table 7.** Experimental environment.

**Internet Connecting Speed**	**100 M bps**
Host CPU	4
Operation System	Windows Server 2008 Enterprise
Virtualized Tool	Hyper-V
RAM	2 G

**Table 8.** Testing results for 4,000/8,000 online users.

**Deploy Host**		**Online Users**	**Time-cost (ms)**	**Total Online Users**	**Total Time-cost (ms)**	**Average users/per ms**
Physical Server	[Test 1] Physical Web Server 2 G	4,000	7.381 ± 1.000	4,000	7.381 ± 1.000	541.93 users/per ms
Physical Server	[Test 2] Virtual Web Server 512 MB	4,000	8.648 ± 1.000	8,000	8.648 ± 1.000	925.07 users/per ms
	[Test 3] Virtual Web Server 1 G	4,000	6.597 ± 1.000			

**Table 9.** Feedback collected from T Company.

**No.**	**Category**	**Feedback**
1	IT aspect	A system based on the logistic cloud can enhance the production process of T Company by supporting a cross-platform system. Old systems in T Company can all use the same platform based on the logistic cloud. Some old systems can be reused based on the logistic cloud.
2	Production aspect	IoT-technology-enabled systems are positive for the operational process of T Company. Inventory process time was reduced by about 20%. The missing rate of products was reduced from 3% to 0.3%. Some employees were delighted to have the platform, which reduced their inventory time.
3	Decision aspect	Real-time production information can be enhanced through the proposed logistic cloud infrastructure. Information is more clear, and it is easier for decision-makers to access. Cost handling and productivity information is easier to estimate.

## References

[1] Nagorny K., Colombo A.W., Schmidtmann U. (2012). A service- and multi-agent-oriented manufacturing automation architecture: An IEC 62264 level 2 compliant implementation. Comput. Ind..

[2] Eliasson J., Kyuasakov R., Delsing J., Nessaether J., Colombo A.W., Jammes F., Karnouskos S., Diedrich C. A migration approach to a SOA-based architecture for next generation process control and monitoring.

[3] Mell P., Grance T. (2009). Effectively and Securely Using the Cloud Computing Paradigm.

[4] Kang S., Myung J., Yeon J., Ha S.-W., Cho T., Chung J.-M., Lee S.-G. (2010). A General Maturity Model and Reference Architecture for SaaS Service. Lect. Note. Comput. Sci..

[5] Atzori L., Iera A., Morabito G. (2010). The internet of things: A survey. Comput. Netw..

[6] Coetzee L., Eksteen J. The internet of things—Promise for the future? An Introduction.

[7] Buratti C., Conti A., Dardari D., Verdone R. (2009). An overview on wireless sensor networks technology and evolution. Sensors.

[8] Sun Z., Li W., Song W., Jiang P. (2011). Research on manufacturing supply chain information platform architecture based on internet of things. Adv. Mater. Res..

[9] Chen S.-L., Chen Y.-Y., Hsu C. (2013). Development of logistic management information system based on web service architecture and RFID technology. Appl. Math. Inf. Sci..

[10] Xu X. (2012). From cloud computing to cloud manufacturing. Robot. Comput. Integr. Manuf..

[11] Li B.H., Zhang L., Wang S.L., Tao F., Cao J.W., Jiang X.D., Song X., Chai X.D. (2010). Cloud manufacturing: A new service-oriented networked manufacturing model. Comput. Integr. Manuf. Syst..

[12] Tao F., Hu Y.F., Zhang L. (2010). Theory and Practice: Optimal Resource Service Allocation in Manufacturing Grid.

[13] Fatahi Valilai O., Houshmand M. (2013). A collaborative and integrated platform to support distributed manufacturing system using a service-oriented approach based on cloud computing paradigm. Robot. Comput. Integr. Manuf..

[14] Hou Z., Zhou X., Gu J., Wang Y., Zhao T. ASAAS: Application software as a service for high performance cloud computing.

[15] Shi J., Li Y., He W., Sim D. (2012). SecTTS: A secure track & trace system for RFID-enabled supply chains. Comput. Ind..

[16] Laili Y., Tao F., Zhang L., Sarker B.R. (2012). A study of optimal allocation of computing resources in cloud manufacturing systems. Int. J. Adv. Manuf. Technol..

[17] Tao F., Zhang L., Venkatesh V.C., Luo Y., Cheng Y. (2011). Cloud manufacturing: A computing and service-oriented manufacturing model. J. Eng. Manuf..

[18] Tao F., LaiLi Y., Xu L., Zhang L. (2013). FC-PACO-RM: A parallel method for service composition optimal-selection in cloud manufacturing system. IEEE Trans. Ind. Inform..

[19] Xu X. (2013). Cloud manufacturing: A new paradigm for manufacturing businesses. Aust. J. Multi-Discip. Eng..

[20] Zhang L., Luo Y., Tao F., Li B.H., Ren L., Zhang X., Guo H., Cheng Y., Hu A., Liu Y. (2014). Cloud manufacturing: A new manufacturing paradigm. Enterp. Inf. Syst..

[21] Wu D., Greer M.J., Rosen D.W., Schaefer D. (2013). Cloud manufacturing: Strategic vision and state-of-the-art. J. Manuf. Syst..

[22] Putnik G. (2012). Advanced manufacturing systems and enterprises: Cloud and ubiquitous manufacturing and an architecture. J. Appl. Eng. Sci..

[23] Chen S.-L., Wang H.P., Chen Y.Y. (2013). Development of software-as-a-service cloud computing architecture for manufacturing management systems based on virtual COM port driver technology. Appl. Mech. Mater..

[24] Giriraj M., Muthu S. (2013). A cloud computing methodology for industrial automation and manufacturing execution system. J. Theor. Appl. Inf. Technol..

[25] Hung M.H., Lin Y.C., Huy T.Q., Yang H.C., Cheng F.T. Development of a cloud-computing-based equipment monitoring system for machine tool industry.

[26] Sun C. (2012). Application of RFID technology for logistics on internet of things. AASRI Procedia.

[27] Lodewijks G., Veeke H.M.P., Lopez de la Cruz A.M. Reliability of RFID in logistic systems.

[28] Tsai W.T. Service-oriented system engineering: A new paradigm.

[29] Langley C.J. (1985). Information-based decision making in logistics management. Int. J. Phys. Distrib. Logist. Manag..

[30] Walker J., Mendelsohn T., Overby C.S. (2004). Vendors Race To Fill A New Void: RFID Middleware. TechStrategy Research Brief.

[31] Jaselskis E.J., Tarek E. (2003). Implementing radio frequency identification in the construction process. J. Constr. Eng. Manag..

[32] Yagi J., Arai E., Arai T. (2005). Co*nst*ruction automation based on parts and packets unification. Int. J. Autom. Constr..

[33] Ergen E., Akinci B., Sacks R. (2007). Tracking and locating components in a precast storage yard utilizing radio frequency identification technology and GPS. Int. J. Autom. Constr..

[34] Wang L. (2008). Enhancing construction quality inspection and management using RFID technology. Autom. Constr..

[35] Chowa Harry K.H., Choya King Lun, Lee W.B., Laub K.C. (2006). Design of a RFID case-based resource management system for warehouse operations. Expert Syst. Appl..

[36] Zhao W., Wang Z., Ku T. A novel large-scale RFID integration Architecture based on SOA for supply chain logistics management.

[37] Kwok S.K., Ting J.S.L., Tsang A.H.C., Lee W.B., Cheung B.C.F. (2010). Design and development of a mobile EPC-RFID-based self-validation system (MESS) for product authentication. Comput. Indust..

[38] Lu K.-Y. (2013). Implementing a Real-Time Shop-Floor Control System Using Zigbee-Based Networking Systems. Int. J. Electron. Business Manag..

[39] Ruiz-Garcia L., Barreiro P.I., Robla J. (2008). Performance of ZigBee-Based wireless sensor nodes for real-time monitoring of fruit logistics. J. Food Eng..

[40] Yu H.C., Zhu H., He F., Wan Y.L. (2012). Design of the remote monitoring system for workshop based on ZigBee wireless sensor networks. Commun. Comput. Inf. Sci..

[41] Chen S.-L., Chen Y.-Y. Design and Implementation of a Global Logistic Tracking System Based on SaaS Cloud Computing Infrastructure.

[42] Chen S.-L., Chen Y.-Y. (2011). Design and implementation of a global logistic tracking system based on SaaS cloud computing infrastructure. J. Syst. Manag. Sci..

[43] Kankanhalli A., Teo H.-H., Tan B.C.Y., Wei K.-K. (2003). An integrative study of information systems security effectiveness. Int. J. Inf. Manag..

